# Semi-analytical modelling and evaluation of uniformly doped silicene nanotransistors for digital logic gates

**DOI:** 10.1371/journal.pone.0253289

**Published:** 2021-06-14

**Authors:** Mu Wen Chuan, Kien Liong Wong, Munawar Agus Riyadi, Afiq Hamzah, Shahrizal Rusli, Nurul Ezaila Alias, Cheng Siong Lim, Michael Loong Peng Tan

**Affiliations:** 1 Faculty of Engineering, School of Electrical Engineering, Universiti Teknologi Malaysia, Skudai, Johor, Malaysia; 2 Department of Electrical Engineering, Diponegoro University, Semarang, Indonesia; University of Glasgow, UNITED KINGDOM

## Abstract

Silicene has attracted remarkable attention in the semiconductor research community due to its silicon (Si) nature. It is predicted as one of the most promising candidates for the next generation nanoelectronic devices. In this paper, an efficient non-iterative technique is employed to create the SPICE models for p-type and n-type uniformly doped silicene field-effect transistors (FETs). The current-voltage characteristics show that the proposed silicene FET models exhibit high on-to-off current ratio under ballistic transport. In order to obtain practical digital logic timing diagrams, a parasitic load capacitance, which is dependent on the interconnect length, is attached at the output terminal of the logic circuits. Furthermore, the key circuit performance metrics, including the propagation delay, average power, power-delay product and energy-delay product of the proposed silicene-based logic gates are extracted and benchmarked with published results. The effects of the interconnect length to the propagation delay and average power are also investigated. The results of this work further envisage the uniformly doped silicene as a promising candidate for future nanoelectronic applications.

## 1. Introduction

Digital logic gates are the foundation of modern computation and information processing in various systems such as nanostructure computers [1,2], photonic technology [3,4] and biomedical engineering [5,6]. The development of these systems is governed by Moore’s Law [7,8] for more than four decades. However, the present digital logic gates, primarily based on bulk silicon (Si) field-effect transistors (FETs), are reaching the fundamental device limitations [9,10]. Therefore, the quest for next-generation FETs to leverage nanotechnologies, for more than Moore’s applications, has become one of the mainstream research topics.

The development of two-dimensional (2D) materials has been motivated by the success of monolayer honeycomb carbon (C)—graphene [11]. While the discovery of graphene has more than 15 years of history, the honeycomb Si-based monolayer—silicene has only attracted research interest in the recent years as shown by the trend of publication numbers [12,13] despite its potential compatibility with the present Si-dominant fabrication processes. In 2015, Tao *et al*. [14] demonstrated the first silicene-based transistor using synthesis-transfer fabrication technique. Interestingly, silicene is envisaged as an alternative material for transistor scaling in the International Roadmap for Devices and Systems (IRDS) [15]. Although silicene sheets have successfully been formed on various substrates in their buckled [16–18] and planar [19] forms, the fabrication of stable free-standing silicene still remains a major challenge. At this early stage of development, computational modelling and simulation are very useful in providing fundamental insights of silicene-based devices and circuits.

Silicene, as a counterpart of graphene [20,21], exhibits the Dirac cone properties and in addition, an extremely small energy bandgap of 1.55 *meV* [22]. Similar to graphene, bandgap engineering techniques are required in order to build silicene-based transistors, where the transistors for digital switching applications typically require an energy bandgap of at least 0.4 *eV* [23] to suppress the unwanted subthreshold conduction. Silicene sheets can be sliced into semiconducting silicene nanoribbons (SiNRs) which have shown promising transistor performances [24], but their energy bandgap values and electronic properties are heavily dependent on the nanoribbons widths [25]. Although altering nanoribbon width is proven to be an viable bandgap engineering option, the fabrication technique to produce nanoribbon with perfect edge control is yet to be discovered, even for the well-known and matured graphene [26].

Due to this shortcoming, we propose to employ the n-type and p-type uniformly doped silicene as the semiconducting channel of the silicene-based FETs, by using phosphorus (P) and aluminium (Al) as the dopant atoms, respectively. This uniform doping technique has been proven previously to be an effective way to obtain semiconducting silicene nanosheets, that are suitable for digital switching applications [27]. The silicene sheets that are uniformly doped using P and Al will be denoted as PSi_3_ and AlSi_3_, respectively in the rest of this paper. Unlike the selective doping technique [28,29], where the electronic properties of doped silicene vary with dopant sites, the uniform doping technique is independent on the dopant sites [30]. After obtaining semiconducting silicene nanosheets, these n-type and p-type silicene-based FETs can be employed to build various complementary metal–oxide–semiconductor (CMOS) circuits such as inverter, NAND, and NOR gates.

**Fig 1:**
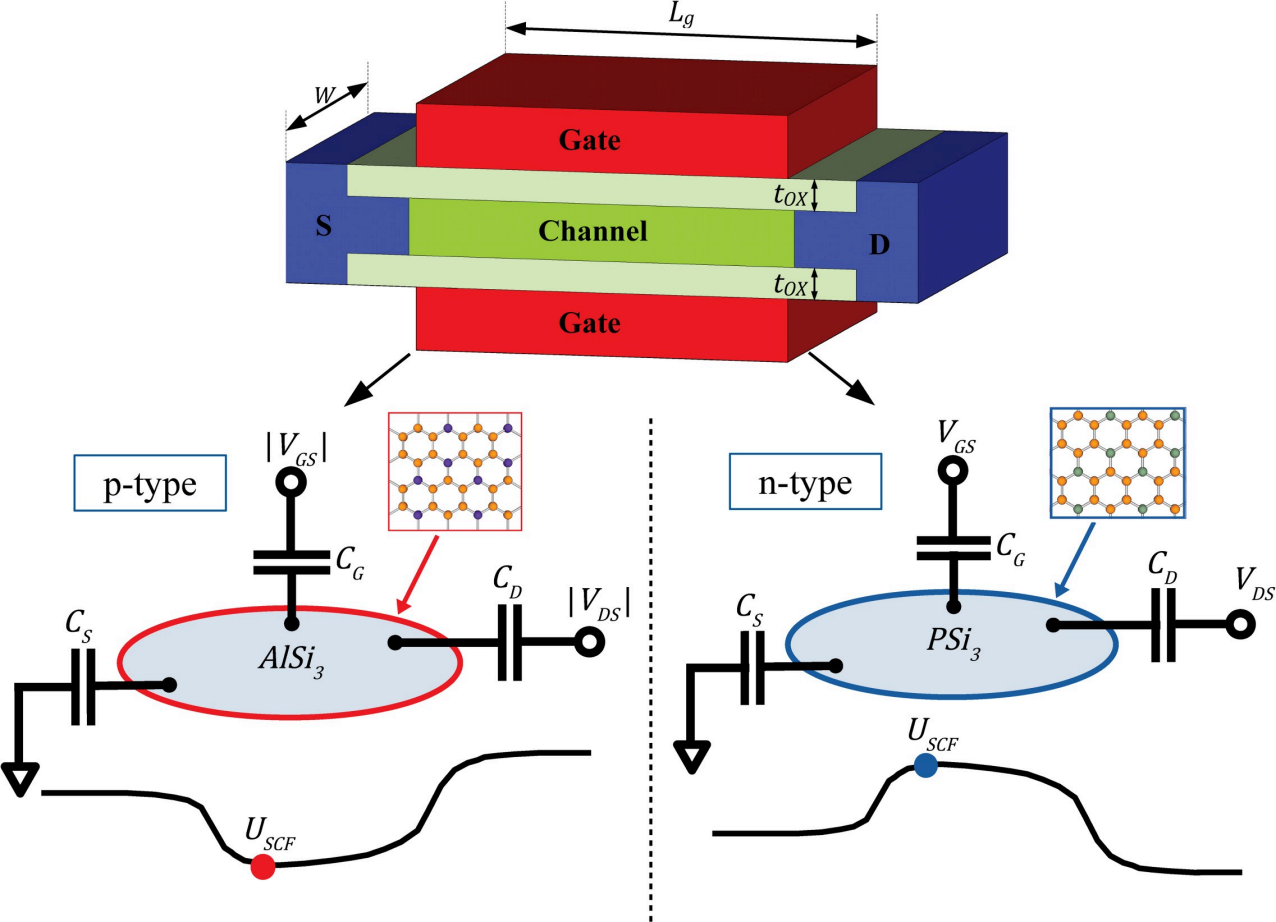
Schematic structure and circuit diagrams of n-type and p-type uniformly doped silicene FETs. Brown atoms represent silicon (Si) atoms; purple atoms represent aluminium (Al) atoms; and grey atoms represent phosphorus (P) atoms.

Despite rigorous efforts to model and simulate silicene-based devices [12,31], there is still minimal work focusing on the circuit-level performance analysis featuring silicene-based transistor. In this paper, the circuit-level performance of n-type and p-type uniformly doped silicene FETs, as shown in **Fig 1**, are assessed by developing a SPICE-compatible model [32]. This circuit-level model is developed by extending our previous works on uniformly doped silicene model at the material-level [33] and device-level [34]. **Section 2** describes the detailed modelling procedures employed in this work. In **Section 3**, the simulation results of the silicene-based logic gates are shown by plotting the timing diagrams. Subsequently, the circuit performance of the logics gates are analysed based on their propagation delay, average power, power-delay product (PDP) and energy-delay product (EDP). Finally, the conclusion of this work is drawn and potential future work is recommended in **Section 4**.

## 2. Modelling procedures

**Fig 2:**
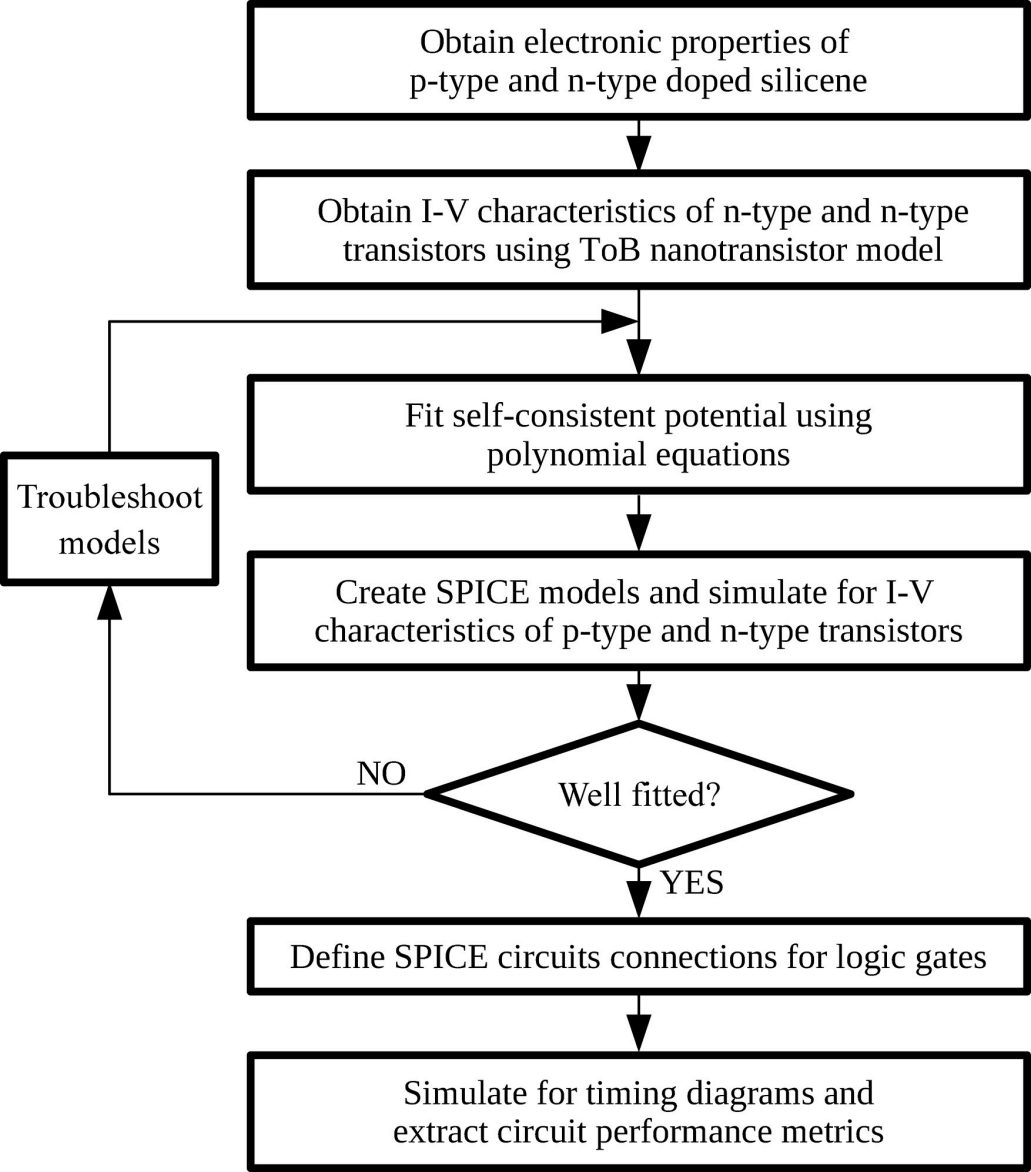
Modelling and simulation flow chart of this work.

This section describes the step-by-step modelling procedures for the proposed silicene-based nanotransistors from material-level up to circuit-level, where the overall flow chart is depicted in **Fig 2**.

### 2.1 Uniformly doped silicene transistors

At the material-level, the electronic properties of p-type (AlSi_3_) and n-type (PSi_3_) nanosheets as shown in **Fig 1**, are modelled using nearest neighbour tight-binding (NNTB) model by fitting the published DFT band structures in **Ref** [27]. The nanosheets are assumed to be in their perfect planar honeycomb lattice. This assumption is applicable because the successful fabrication of planar silicene has recently been reported [19]. Subsequently, the transverse effective masses (in the zigzag direction) of these materials are obtained by using parabolic band approximation. The detailed procedures to obtain the effective masses can be found in **Ref** [33]. Although previous work [33] shows only the modelling procedures for the p-type AlSi_3_ nanosheet, the same technique is repeated to compute the electronic properties of n-type PSi_3_ nanosheet, in order to obtain both type of transistors for CMOS applications. **Table 1** summarises the important electronic properties of the AlSi_3_ and PSi_3_ nanosheets, where *m*_0_ is the constant for electron rest mass. The results show that both uniformly doped silicene nanosheets have achieve energy bandgap values of 0.4 *eV*≤*E*_*g*_≤3.0 *eV*, making them suitable for nanoelectronic digital switching applications [35].

**Table 1:** Electronic properties of uniformly doped silicene nanosheets.

Parameters	AlSi_3_	PSi_3_
**Semiconductor type**	p-type	n-type
**Energy bandgap, *E*_*g*_ (*eV*)**	0.780	0.660
**Electron effective mass, $m_{e}^{*}$**	0.235*m*_0_	0.230*m*_0_
**Hole effective mass, $m_{h}^{*}$**	0.255*m*_0_	0.240*m*_0_

With the obtained electronic properties, the work then proceeds with device-level modelling by employing the top-of-the-barrier (TOB) ballistic nanotransistor model [36], which has been widely used to predict the performance limits of various low-dimensional materials [37]. In this work, a double-gated FET structure with *L*_*g*_ = 10 *nm* is employed, where the gate oxide layers are SiO_2_ (*ε*_*r*_ = 3.9) with thickness of *t*_*OX*_ = 1.5 *nm*. All simulations are done at the room temperature of *T* = 300 *K*. For simplification, the *W*/*L*_*g*_ aspect ratio is set as unity for both type of silicene FETs. The schematic diagrams of the FETs are illustrated in **Fig 1**. In n-type 2D FETs, the current transport primarily depends on the electron mobility via the conduction band, and vice versa for p-type 2D FETs (where the structures of 2D FETs are similar to those of junctionless FETs [38]). Therefore, electron effective mass is used for n-type (PSi_3_) FET while hole effective mass is used for p-type (AlSi_3_) FET.

**Fig 3:**
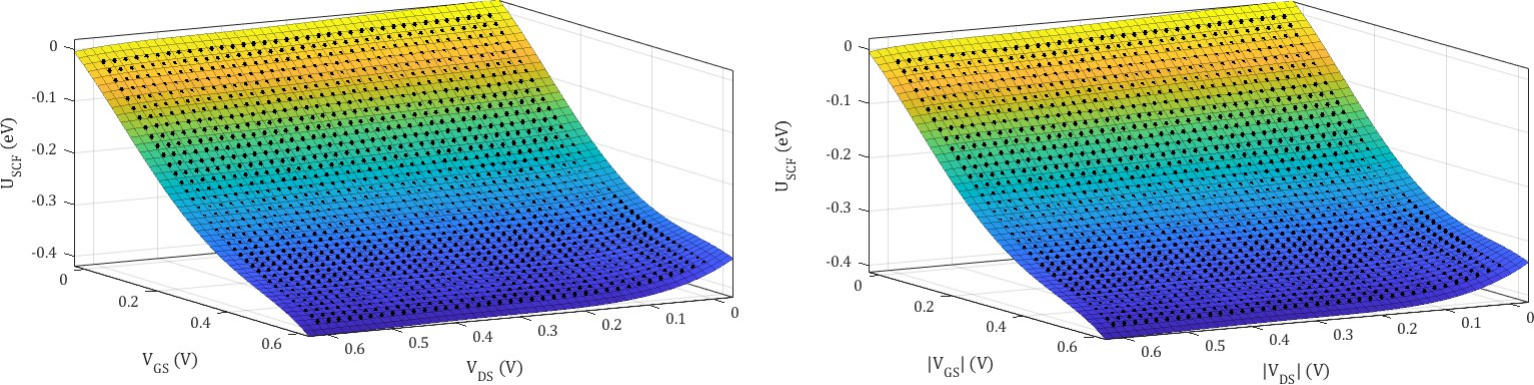
The non-linear regression fit for self-consistent potential: (a) p-type AlSi_3_ and (b) n-type PSi_3_. The dots are *U*_*SCF*_ data from ToB model; and the coloured plane is the plot of fifth order polynomial equation.

The self-consistent potentials *U*_*SCF*_ in the TOB model are calculated iteratively in MATLAB until the solutions for the charge carriers at the TOB converge. Therefore, further modifications are required to obtain a non-iterative model, in terms of the drain-source voltage *V*_*DS*_ and gate-source voltage *V*_*GS*_, to allow cross-platform simulation and reduce computational cost [39]. In order to create a model compatible with the Simulation Program with Integrated Circuit Emphasis (SPICE), the self-consistent potential *U*_*SCF*_ in the TOB model is computed through fifth order polynomial equation within the non-linear regression model [40], expressed as

$$U_{SCF}(\left|V_{GS}|,|V_{DS}\right|)=P_{ij}\sum_{k=0}^{5}{\left(|V_{GS}|+|V_{DS}|\right)}^{k},$$ (1)

where *P*_*ij*_ is the coefficient for each respective |*VG*_*S*_|^*i*^|*V*_*DS*_|^*j*^ term. The fifth order binomial equation as shown in **Eq (1)** can be expanded via Pascal’s triangle rule. The coefficients are extracted and optimised using MATLAB curve fitting tool, where **Fig 3** shows the results of the non-linear regression model. The full equation and coefficients for p-type and n-type uniformly doped silicene is attached in the supplementary data file (**S1 File**). We employed the fifth order binomial equation because the lower order binomial equations are unable to produce decent fit for the *U*_*SCF*_ and the fifth order is the highest available within the curve fitting tool.

### 2.2 Device and circuit simulation

Following that, the current-voltage (I-V) characteristics of n-type and p-type uniformly doped silicene FETs can be obtained in terms of *V*_*DS*_ and *V*_*GS*_, by using Landauer-Büttiker ballistic transport equation [36] with Fermi-Dirac integral solution [41], given as

$$|I_{DS}(\left|V_{GS}|,|V_{DS}\right|)|=\frac{gW}{\hbar^{2}}\sqrt{\frac{{m_{x}^{*}q^{2}(k_{B}T)}^{3}}{2\pi^{3}}}\left\{\log\left[1+e^{\eta_{S}(\left|V_{GS}|,|V_{DS}\right|)}\right]-\log\left[1+e^{\eta_{D}(\left|V_{GS}|,|V_{DS}\right|)}\right]\right\},$$ (2)

with the normalised source and drain energies of

$$\eta_{S}(\left|V_{GS}|,|V_{DS}\right|)=\frac{E_{F}-U_{SCF}(|V_{GS}|,|V_{DS}|)}{k_{B}T},$$ (3)

and

$$\eta_{D}(\left|V_{GS}|,|V_{DS}\right|)=\frac{E_{F}-U_{SCF}(|V_{GS}|,|V_{DS}|)-q|V_{DS}|}{k_{B}T},$$ (4)

where *g* is the degeneracy factor (set as 2 to include up and down spins); ℏ is the Planck’s constant; *q* is the constant for electric charge; and *k*_*B*_ is the Boltzmann constant.

The magnitude of the supply voltage |*V*_*DD*_| proposed in this work is 0.60 *V* and the Fermi energy level *E*_*F*_ is adjusted such that the off-current *I*_*off*_ = 100 *nA*/*μm*, for low-standby power (LSTP) CMOS applications [42]. In this work, the original gate control (*α*_*D*_ = 0.880) and drain control (*α*_*D*_ = 0.035) parameters from **Ref** [36] are employed and the source terminal is always tied to the ground (*V*_*S*_ = 0 *V*). Eqs **(1)** to **(4)** are used to create the SPICE library files for n-type and p-type uniformly doped silicene FET. With these operating conditions, the n-type and p-type silicene FETs have achieved on-to-off current (*I*_*on*_/*I*_*off*_) ratio of *I*_*on*_/*I*_*off*_ = 2.8×10^5^ and *I*_*on*_/*I*_*off*_ = 2.6×10^5^, respectively at room temperature *T* = 300 *K*. The n-type silicene FET has higher *I*_*on*_/*I*_*off*_ ratio because the electron effective mass of PSi_3_ nanosheet is lighter than the hole effective mass of AlSi_3_. The *I*_*on*_/*I*_*off*_ ratio of the proposed device is higher than Si FinFET [43] by two orders. In addition, the *I*_*on*_/*I*_*off*_ ratios of the n-type and p-type uniformly doped silicene FETs are improved by 35.7% and 19.2%, respectively, when compared to n-type and p-type Si nanowire FETs (where the *I*_*on*_/*I*_*off*_ ratios of n-type and p-type Si nanowire FETs were found to be 1.8×10^5^ and 2.1×10^5^, respectively in **Ref** [44]). In addition, **Table 2** compares the *I*_*on*_/*I*_*off*_ ratios of the proposed model with published 2D material-based FET models. It is shown that the proposed FET models outperform Phosphorene and graphene nanoribbon (GNR) FET models. Although 27-ASiNR FET outperforms the proposed FETs, it still remains a huge challenge to precisely control the size of 2D nanoribbons with specific widths, even for graphene which was discovered in the laboratory more than 15 years ago [26,45].

**Table 2:** Comparison of *I*_*on*_/*I*_*off*_ ratio of the proposed model with published 2D material-based FET models.

[Ref]	Channel	L (nm)	Gate oxide	t_OX_ (nm)	I_on_/I_off_ ratio
This work	PSi_3_	10	SiO_2_	1.5	2.8×10^5^
This work	AlSi_3_	10	SiO_2_	1.5	2.6×10^5^
[24]	27-ASiNR	15	-	1.0	2.8×10^6^
[42]	Phosphorene	20	ZrO_2_	3.0	1.0×10^4^
[46]	GNR	10	Mixed	1.5	4.5×10^4^

**Fig 4:**
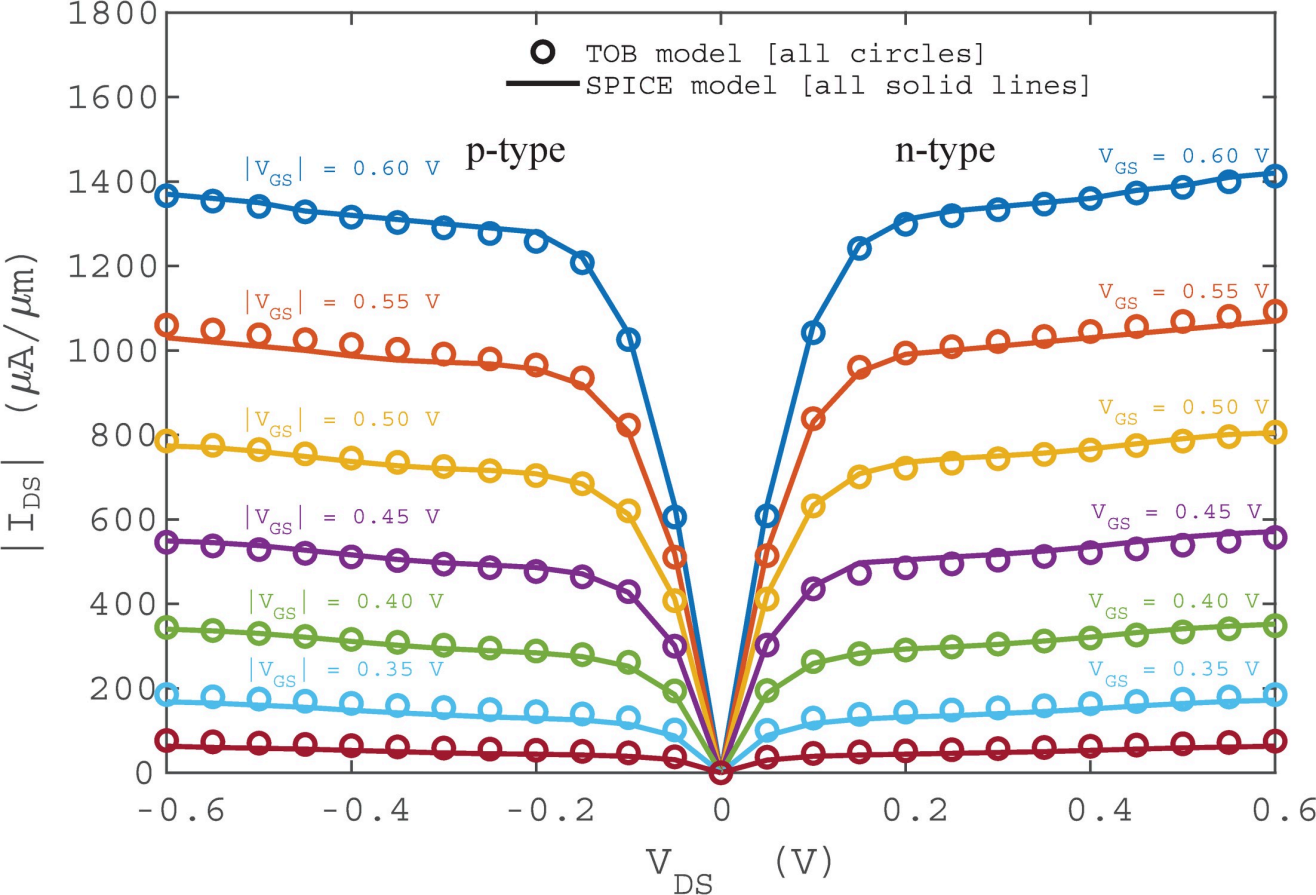
Comparison of I-V characteristics between iterative and non-iterative TOB nanotransistor models.

The simulated I-V characteristics for the original iterative TOB model and non-iterative SPICE model are plotted on the same graph in **Fig 4**. The results show that the fifth order binomial equation for *U*_*SCF*_ is capable of reproducing the iterative TOB model in the HSPICE circuit simulator with minimal error. With the p-type and n-type FET SPICE models ready, the work proceeds to build and simulate digital logic circuits using HSPICE simulator. In order to make the circuit simulation more practical, copper (Cu) interconnect capacitance is incorporated as the load capacitance for all circuits. The Cu interconnect capacitance is identified as *C*_*int*_ = 121.3 *aF*/*μm* by using the ITRS projected interconnect capacitance value for transistor with 10 *nm* gate length [40]. The length *L*_*int*_ of the Cu interconnect is varied from 10 *nm* to 50 *μm* to investigate its effects on the logic circuit performance.

## 3. Results and discussions

In this section, the simulation results of digital logic gates, including inverter, 2-input NAND and 2-input NOR gates are shown. Their circuit performances are also evaluated by extracting the propagation delay (*t*_*p*_), average power (*P*_*avg*_), PDP and EDP. The propagation delay of the proposed model is also benchmarked with selected published results.

### 3.1 Timing diagrams

**Fig 5:**
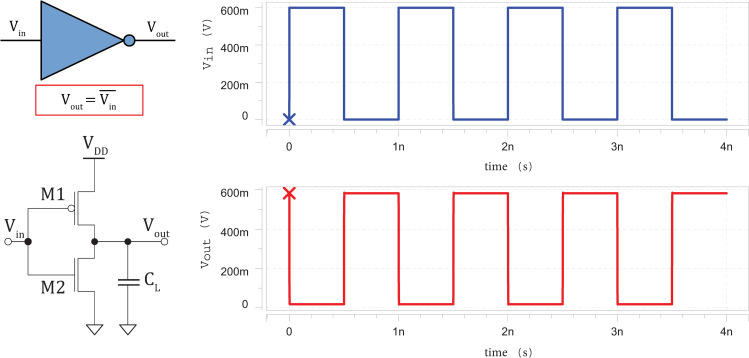
Schematic circuit diagram of silicene-based inverter (*L*_*int*_ = 1 *μm*) with its input (blue) and corresponding output waveforms (red).

**Fig 6:**
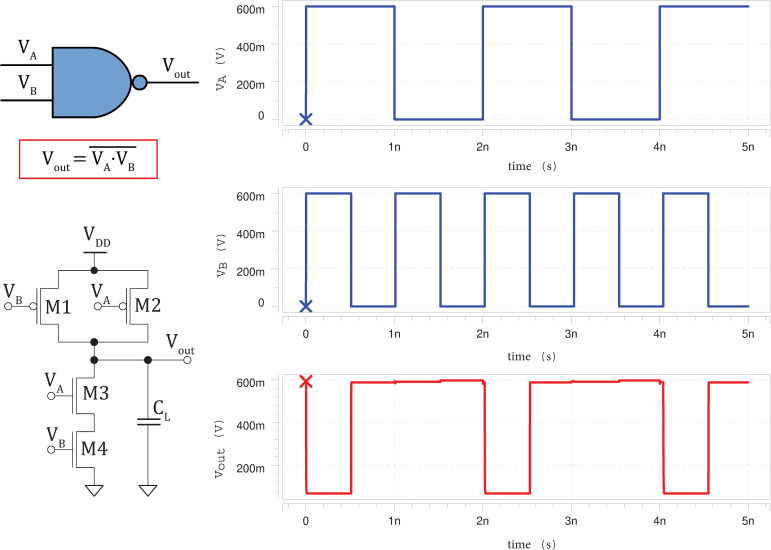
Schematic circuit diagram of silicene-based 2-input NAND gate (*L*_*int*_ = 1 *μm*) with its input (blue) and corresponding output waveforms (red).

**Fig 7:**
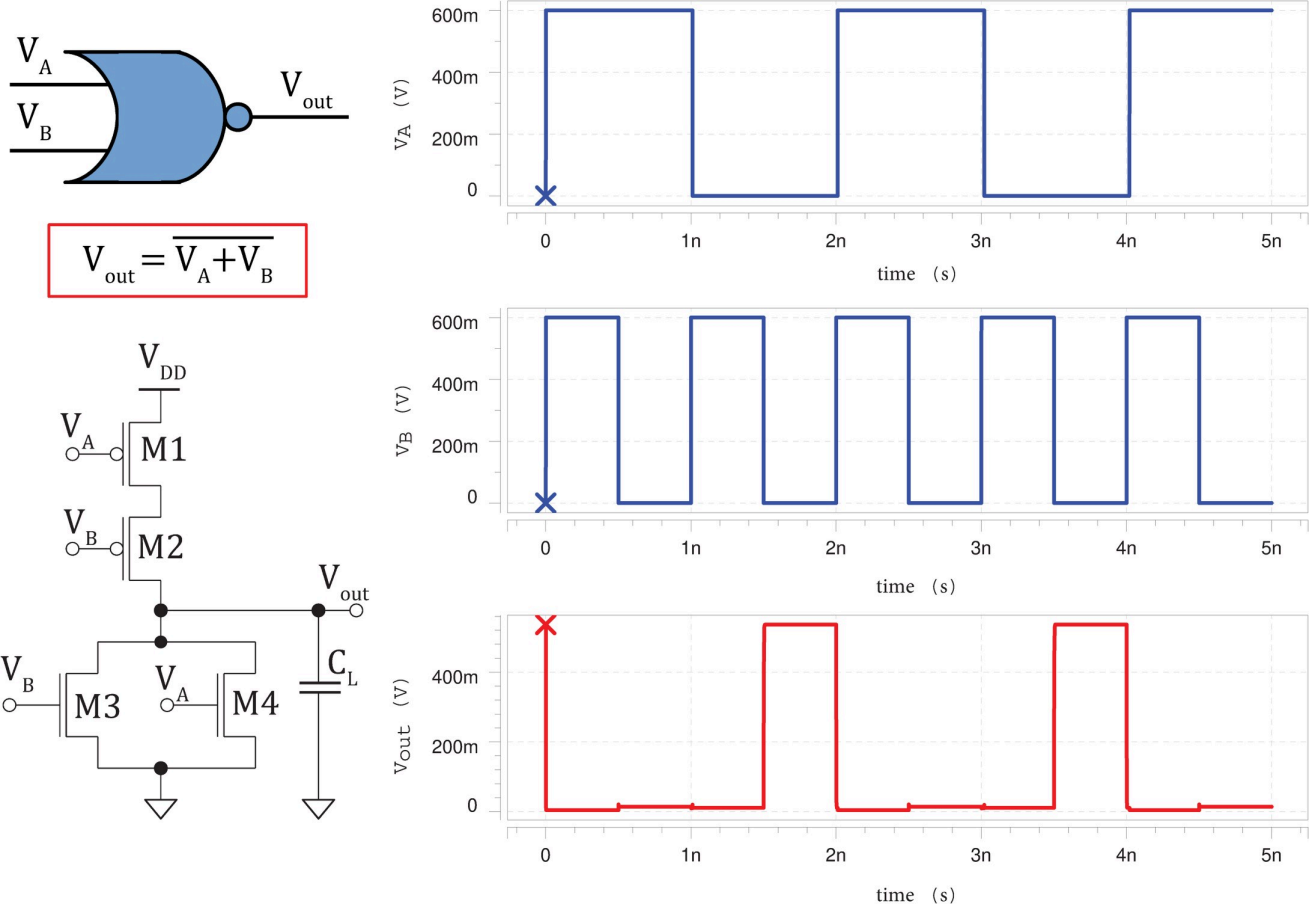
Schematic circuit diagram of silicene-based 2-input NOR gate (*L*_*int*_ = 1 *μm*) with its input (blue) and corresponding output waveforms (red).

The silicene-based logic circuits simulated in HSPICE are then plotted using Avanwaves. The high voltage (representing ‘1’ digital signal) of the input pulses are set to the supply voltage of 0.60 *V*; and low voltage (representing ‘0’ digital signal) of the input pulses are set to the ground voltage of 0 *V*. A rise and fall time of *t*_*r*_ = *t*_*f*_ = 0.1 *ps* are used for the input waveforms in order to obtain sharp rising and falling edges. **Figs 5**–**7** clearly show that the silicene-based logic circuits are able to function correctly according to the intended Boolean logics for inverter, 2-input NAND and 2-input NOR gates [47], respectively.

### 3.2 Performance analysis of digital logic circuits

**Fig 8:**
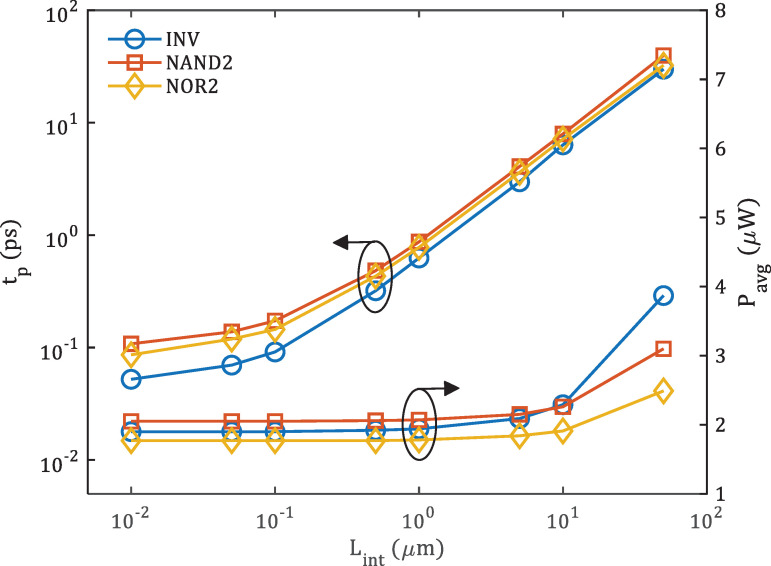
Propagation delay (*t*_*p*_) and average power (*P*_*avg*_) of silicene-based digital logic circuits with varying interconnect length (*L*_*int*_). INV, NAND2 and NOR2 represent inverter, 2-input NAND and 2-input NOR gates, respectively.

The propagation delay (*t*_*p*_) and average power (*P*_*avg*_) for the simulated logic gates are extracted and plotted against the length *L*_*int*_ of Cu interconnect, as depicted in **Fig 8**. It is clearly shown that the *t*_*p*_ for all three logic gates increases as the *L*_*int*_ increases. Nevertheless, the 2-input NAND gate has the highest *t*_*p*_ for all interconnect lengths. On the other hand, the *P*_*avg*_ for all three logic gates remain almost constant until *L*_*int*_ = 1 *μm*, regardless of the type of logic gates. Thus, it is crucial to optimise the *L*_*int*_ in digital system design in order to achieve minimal propagation delay and suppress the power consumption. Similar circuit degradation due to long *L*_*int*_ was also previously reported for GNR FETs with interconnect analysis [40].

**Fig 9:**
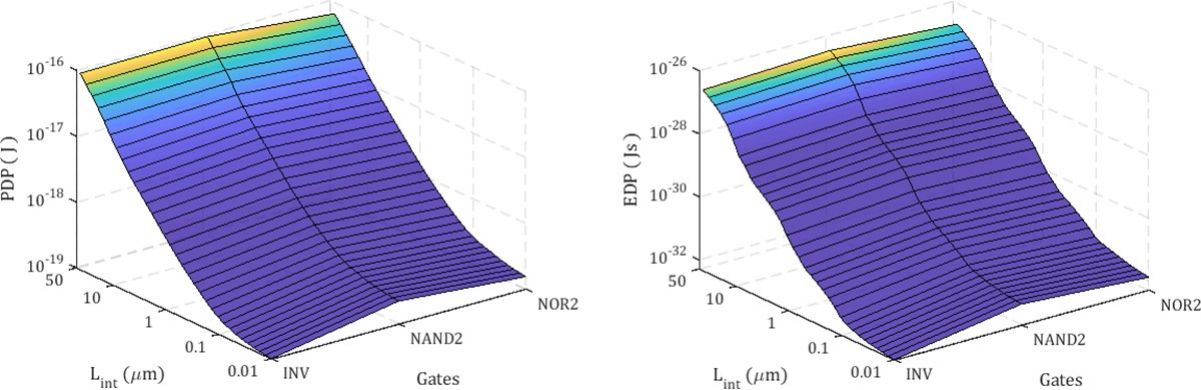
(a) PDP and (b) EDP of silicene-based digital logic circuits. INV, NAND2 and NOR2 represent inverter, 2-input NAND and 2-input NOR gates, respectively.

Subsequently, the figure of merits for digital logic circuits are calculated using the extracted values in **Fig 8** and the equations of the PDP and EDP [47], given as

$$PDP=P_{avg}\times t_{p},$$ (5)

and

$$EDP=PDP\times t_{p},$$ (6)

where the average power *P*_*avg*_ and propagation delay *t*_*p*_. **Fig 9** shows the PDP and EDP of proposed silicene-based digital logic circuits when *L*_*int*_ is varied from 10 *nm* to 50 *μm*. The 3D plot in **Fig 9(b)** show that, at all values of *L*_*int*_, 2-input NAND gate has the highest EDP due to its high propagation delay *t*_*p*_ compared to inverter and 2-input NOR gates.

**Fig 10:**
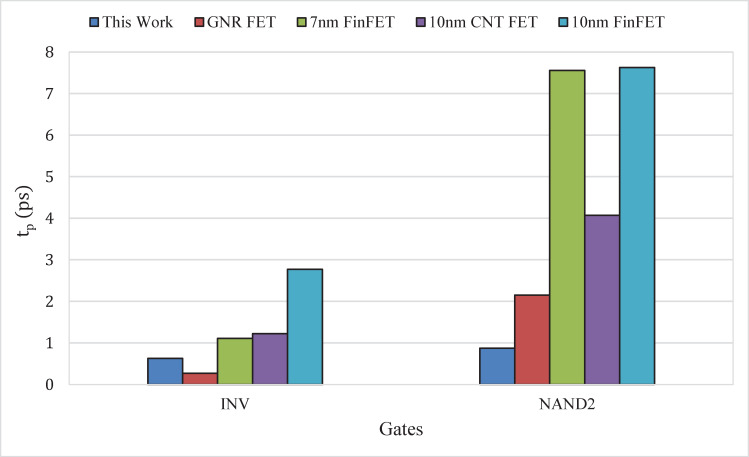
Comparison of propagation delay (*t*_*p*_) between the proposed silicene-based logic circuit with recent published results. INV and NAND2 represent inverter and 2-input NAND gates, respectively.

As this study aims to assess the circuit-level performance of the proposed uniformly doped silicene FETs for digital logic gates, the results are benchmarked with published works that are based on low-dimensional materials, including GNR FET and 7 *nm* FinFET from **Ref** [48]; as well as 10 *nm* carbon nanotube (CNT) FET and 10 *nm* FinFET from **Ref** [49]. Due to the unavailability of complete data, we have only compared the propagation delay *t*_*p*_ among the models for inverter and 2-input NAND gates as shown in **Fig 10**. The bar graph clearly shows that the proposed silicene-based inverter gate outperforms all the published models in terms of the propagation delay. However, the propagation delay of proposed silicene-based 2-input NAND gate is higher than that of the graphene-based logic circuit [48]. Despite this slight disadvantage, silicene-based circuits are still a prospective choice for the future nanoelectronic applications due to its potential compatibility with Si CMOS technology [14].

## 4. Conclusions

In this paper, we have investigated the circuit-level performance of digital logic gates built using the p-type and n-type uniformly doped silicene FETs. By fitting the self-consistent potential at the TOB using fifth order binomial equations, a non-iterative SPICE model for the proposed FETs are created, where the model is then utilised to perform circuit-level simulations. Following that, the timing diagrams for the proposed silicene-based logic gates are computed and verified. In order to gain more insights from the digital logic output waveforms, the figure of merits for inverter, 2-input NAND, and 2-input NOR gates are extracted and compared to recent published results. Based on the benchmark of the results, the proposed silicene-based inverter has achieved the lowest propagation delay. Although the propagation delay of the proposed silicene-based 2-input NAND gate is outperformed by GNR-based gate, it is still optimistic that silicene-based CMOS logic circuits are promising substitutes for future nanoelectronic devices because graphene-based systems might require an entirely redesigned fabrication technique and equipment for mass production in the semiconductor industry. In future work, it may be useful to extend the present study on the basic logic gates to explore more complex silicene-based digital circuits and systems.

## Supporting information

S1 File Equations and coefficients of self-consistent potential. (DOCX)
